# Genome-wide association study for somatic cell score in Valdostana Red Pied cattle breed using pooled DNA

**DOI:** 10.1186/s12863-014-0106-7

**Published:** 2014-10-06

**Authors:** Maria G Strillacci, Erika Frigo, Fausta Schiavini, Antonia B Samoré, Fabiola Canavesi, Mario Vevey, Maria C Cozzi, Morris Soller, Ehud Lipkin, Alessandro Bagnato

**Affiliations:** Department of Health, Animal Science and Food Safety (VESPA), University of Milan, Via Celoria 10, 20133 Milan, Italy; Dipartimento di Scienze e Tecnologie Agro - Alimentari, University of Bologna, Viale Fanin 46, 40127 Bologna, Italy; Department of Genetics, The Hebrew University of Jerusalem, 91904 Jerusalem, Israel; Associazione Nazionale Allevatori Bovini di Razza Valdostana (A.N.A.Bo.Ra.Va.), Fraz. Favret, 5, 11020 Gressan, AO Italy

**Keywords:** Mastitis, GWAS, Valdostana Red Pied breed, SCS

## Abstract

**Background:**

Mastitis is a major disease of dairy cattle occurring in response to environmental exposure to infective agents with a great economic impact on dairy industry. Somatic cell count (SCC) and its log transformation in somatic cell score (SCS) are traits that have been used as indirect measures of resistance to mastitis for decades in selective breeding. A selective DNA pooling (SDP) approach was applied to identify Quantitative Trait Loci (QTL) for SCS in Valdostana Red Pied cattle using the Illumina Bovine HD BeadChip.

**Results:**

A total of 171 SNPs reached the genome-wide significance for association with SCS. Fifty-two SNPs were annotated within genes, some of those involved in the immune response to mastitis. On BTAs 1, 2, 3, 4, 9, 13, 15, 17, 21 and 22 the largest number of markers in association to the trait was found. These regions identified novel genomic regions related to mastitis (1-Mb SNP windows) and confirmed those already mapped. The largest number of significant SNPs exceeding the threshold for genome-wide significant signal was found on BTA 15, located at 50.43-51.63 Mb.

**Conclusions:**

The genomic regions identified in this study contribute to a better understanding of the genetic control of the mastitis immune response in cattle and may allow the inclusion of more detailed QTL information in selection programs.

**Electronic supplementary material:**

The online version of this article (doi:10.1186/s12863-014-0106-7) contains supplementary material, which is available to authorized users.

## Background

Mastitis is one of the most frequent inflammatory disease with a significant economic implication for the dairy herds and the resistance to this pathology may be improved by breeding.

The development of mastitis is the result of the interaction among three components: the individual genotype, the pathogens (ordinarily classified in contagious and environmental bacteria) and the environment (hygiene, housing, climate, milking machines, feeding) [1].

The resistance to an infection disease or the absence of susceptibility may be defined as the immune response ability (immuno-competence capability) of an animal, to avoid the pathogens replication after the establishment of an infection. This implies that animals tend to vary in their genetic potential for immuno-competence [2]. The genetic resistance or the genetic susceptibility to mastitis involves interlinked biological mechanisms that activate and regulate the different levels of the immune response, as a consequence of the differences existing in the response to mastitis involving several pathogens [3]. A better understanding of the immune system and of the metabolic pathways involved in the response to various pathogens of resistant and susceptible animals may be used as complementary approach for the disease control.

The discovery of millions of SNP markers in animal genomes forming dense marker panels, and the concomitant decrease in genotyping costs have allowed the performing of genome-wide association studies (GWAS) [4]. The availability of SNP dense genotypes have increased the power of the identification of QTL related to the traits of interest [5], allowing more accurate breeding values estimation with the use of genomic selection methodology and helping the understanding of the genetic control of the traits of interest [6]. Because of the established knowledge of the positive genetic correlation between clinical mastitis and SCS ranging from 0.6 to 0.8 [1], SCC is one of the traits used as an indirect measure of mastitis resistance/susceptibility in breeding programs in cattle and sheep. Many GWAS have detected QTL for SCC in cattle on BTAs 5, 6, 8, 11, 17, 18, 20 and 23 in cosmopolite improved dairy cattle breeds [1,7].

The high costs of screening large populations for marker allele frequencies can be decreased using the SDP approach, genotyping pooled DNA samples from selected individuals at each of the two phenotypic extremes of the trait distribution [8]. Equal amounts of DNA are pooled from individuals in the extreme tails, and pools are then genotyped to estimate allele frequency differences for each SNP among high and low tail pools. The significant identified candidate SNPs are then used for confirmatory association studies [9].

The aim of this study was to identify QTL associated with SCS as an indicator of mastitis. We performed a GWA study for SCS in the Valdostana Red Pied cattle, with a selective DNA pooling analysis, using the Illumina BovineHD Bead chip.

## Results and discussion

Among the 2,417 bulls with DP-EBV values, 275 had semen samples available in the Valdostana Red Pied bio-bank that encompassed in total 373 sires samples spanning across generations.

The Valdostana Red Pied population counting at present about 11,000 milking cows, did not undergo focused selection for milk production only and no gene introgression from other populations have ever occurred. The breed is strongly adapted to harsh alpine environment because breed natural adaptation and because has been selected to maintain pasture capability (summer pasture is the common farming system), longevity, functionality and fertility. Thus the population is somehow a unique genetic resource to map mastitis resistance, a trait related to adaptation, functionality and longevity. The study used all the sire samples available in the Valdostana Red Pied bio-bank thus highlighting the overall observable variability for productive and functional traits in this breed. The smaller number of sires available for the study respect to mapping, may limit the capacity to disclose QTL for mastitis resistance. Nevertheless the experimental design here used and the genetic makeup of the population allowed to identify several new QTL and confirm regions identified in the Italian and Swiss Brown population [10], another breed originating from alpine region, now strongly selected for milk production.

Descriptive statistics for the DP-EBVs and the size of the pools for each tail are reported in Table 1.

**Table 1:** **Details for DP-EBVs mean and their SD values and DP-EBVs reliability (REL) values for low and high tail pools**

**Pool**	**N° of samples**	**DP-EBV mean**	**Mean SD**	**DP-EBV REL mean**	**Pool**	**N° of samples**	**DP-EBV mean**	**Mean SD**	**DP-EBV REL mean**
Low tail_1	20	−1.151	0.324	0.535	High tail_1	20	1.257	0.395	0.493
Low tail_2	19	−1.080	0.251	0.600	High tail_2	20	1.134	0.285	0.574

The initial dataset included 721,644 SNPs. After editing, the association analysis were performed with 655,665 SNPs for SCS DP-EBV.

**Figure 1:**
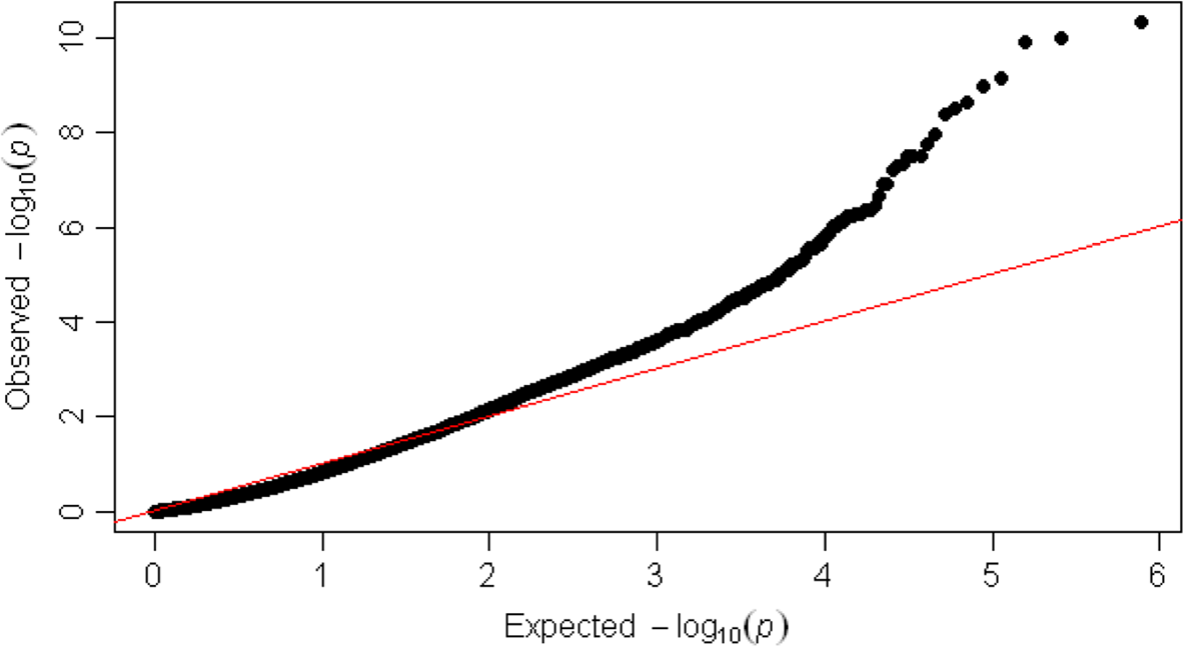
**Q-Q plot of SNPs at marker level (p-values).**

**Figure 2:**
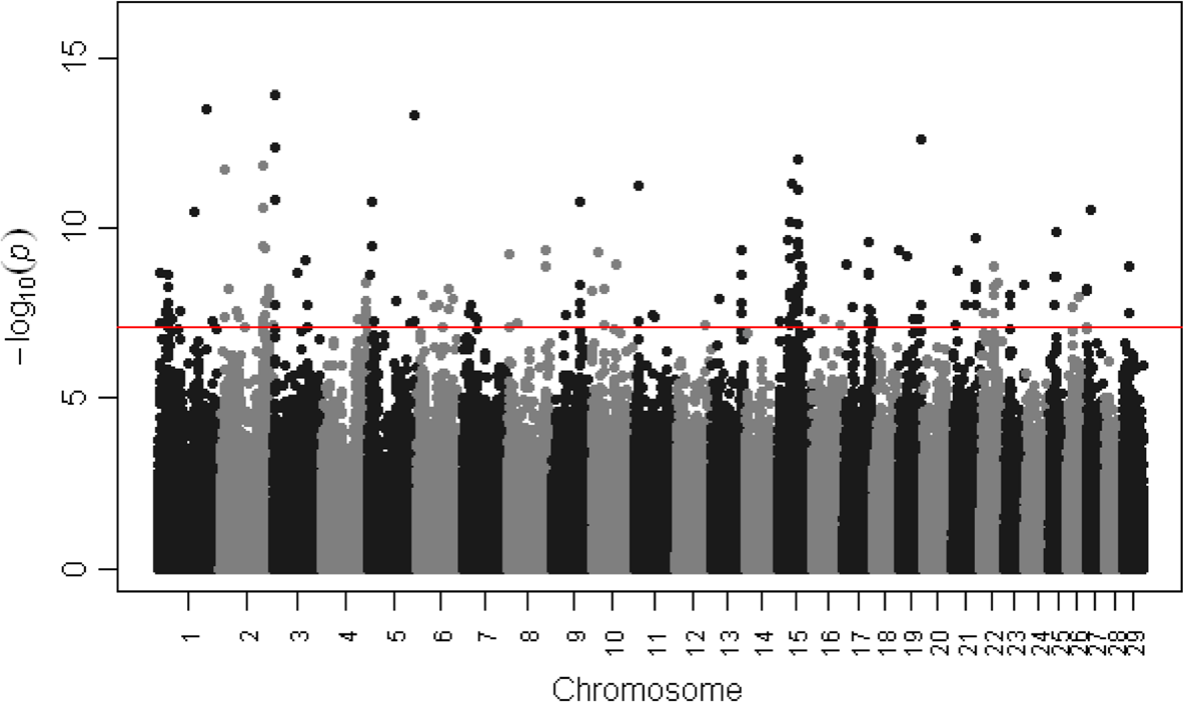
**Manhattan plot of genome-wide associations for SCS trait.** The red line represents the Bonferroni correction threshold.

Figure 1 shows the Q-Q plot of SNPs at marker level (p-values). Deviations from the identity line showed the amount of false positive tests resulted from the analysis of the data. Figure 2 showed the Manhattan plot of genome-wide associations for SCS trait.

A total of 171 significant SNPs in 24 chromosomes were identified above the Bonferroni genome-wide threshold of 0.05. The Additional file 1 showed the list of the 171 significant SNPs identified. The SNPs location and the gene annotation were reported for both the UMD3.1 and Btau4.6.1 assembly. Table in Additional file 1 included the indication of QTL, amongst the ones here disclosed, reported in the online AnimalQTLdb (http://www.animalgenome.org/cgi-bin/QTLdb/index) for clinical mastitis, SCC and SCS.

### Intragenic SNPs

Among the 171 significant markers, 52 SNPs were annotated within 36 genes (Table 2). In Table 2 the significant intragenic SNPs and their corresponding annotated genes in the Btau4.6.1 assembly are reported.

**Table 2:** **Significant intragenic SNPs above the Bonferroni genome-wide threshold of 0.05**

**Illumina SNP name**	**Genbank SNP code**	**P-Value**	**BTA**	**SNP location**	**Gene symbol**
*BovineHD0100007623*	*rs137585939*	4.43E-08	1	26254309	*ROBO1*
*BovineHD0100040084*	*rs43273786*	5.41E-08	1	141228619	*NEK11*
*BovineHD0200004154*	*rs110997154*	3.76E-08	2	15142189	*SSFA2*
*BovineHD0300000560*	*rs110459674*	inf	3	2612333	*TADA1*
*BovineHD0300002104*	*rs110093914*	1.87E-08	3	7276675	*DDR2*
*BovineHD0300018913*	*rs42371455*	1.87E-09	3	66866123	*LPHN2*
*BovineHD0300023699*	*rs135870054*	8.96E-10	3	87296944	*ALG6*
*BovineHD0400026934*	*rs109307332*	4.69E-08	4	98543003	*PLXNA4*
*BovineHD0500003126*	*rs134685896*	3.26E-10	5	12834395	*ACSS3*
*BovineHD0700010213*	*rs133885406*	4.73E-08	7	33526558	*HSD17B4*
*BovineHD0900019961*	*rs136413030*	3.70E-08	9	73355572	*VNN1*
*BovineHD1000004333*	*rs43612234*	5.32E-10	10	12722576	*MEGF11*
*BovineHD1000009424*	*rs43623003*	6.65E-08	10	28079552	*MIR2284Z-1*
*BovineHD1000009428*	*rs110034517*	5.56E-09	10	28102288	*MIR2284Z-1*
*BovineHD1000017503*	*rs42486408*	1.16E-09	10	60793897	*TRPM7*
*BovineHD1100003814*	*rs109489659*	5.53E-12	11	11771322	*CCT7*
*BovineHD1300006368*	*rs109943824*	1.12E-08	13	20845530	*PLXDC2*
*BovineHD1300022672*	*rs41710487*	4.53E-10	13	78416778	*KCNB1*
*BovineHD1500008135*	*rs134980659*	6.49E-08	15	28399876	*THY1*
*Hapmap40064-BTA-36665*	*rs41631137*	4.65E-12	15	33953859	*PIK3C2A*
*BovineHD1500015036*	*rs41769292*	5.15E-09	15	50730325	*NUP98*
*BovineHD1500015037*	*rs134338365*	2.50E-10	15	50733648	*NUP98*
*BTB-00604170*	*rs41769258*	5.84E-10	15	50753778	*NUP98*
*BovineHD1500015042*	*rs41769237*	3.41E-10	15	50765770	*NUP98*
*BovineHD1500015044*	*rs109649273*	6.55E-08	15	50769861	*NUP98*
*BovineHD1500015047*	*rs41768429*	7.66E-12	15	50774198	*NUP98*
*BovineHD1500015049*	*rs41768423*	1.42E-08	15	50780537	*NUP98*
*BovineHD1500015051*	*rs41768414*	6.94E-11	15	50784307	*NUP98*
*BovineHD1500015054*	*rs41768364*	7.12E-08	15	50792403	*NUP98*
*BovineHD1500015055*	*rs109966062*	1.99E-08	15	50795681	*NUP98*
*BovineHD1500015056*	*rs41768379*	9.17E-13	15	50799229	*NUP98*
*BovineHD4100012071*	*rs136525289*	7.93E-09	15	51638163	*PDE2A*
*BTA-18105-no-rs*	*rs109715014*	4.33E-09	15	62952170	*CCDC73*
*BovineHD1600009946*	*rs41798963*	4.62E-08	16	31290905	*CEP170*
*BovineHD1600021693*	*rs41819133*	7.33E-08	16	71743691	*CAMK1G*
*BovineHD1700002750*	*rs110828704*	1.11E-09	17	10472292	*NR3C2*
*BovineHD1700018352*	*rs135157738*	2.29E-09	17	64466089	*RPH3A*
*BovineHD1700019237*	*rs110644998*	2.43E-10	17	67344705	*CORO1C*
*BovineHD1700019238*	*rs134453171*	1.90E-09	17	67347843	*CORO1C*
*BovineHD1700020721*	*rs109085689*	4.61E-08	17	72364594	*MTMR3*
*BovineHD1700021131*	*rs135044766*	4.88E-08	17	73638738	*DEPDC5*
*BovineHD1700021132*	*rs135814317*	7.10E-08	17	73640453	*DEPDC5*
*BovineHD1900006167*	*rs134967563*	7.01E-10	19	20731168	*SSH2*
*BovineHD4100014346*	*rs29017164*	2.41E-13	19	57590345	*ATP5H*
*BovineHD2100001405*	*rs133992914*	6.47E-08	21	6826694	*IGF1R*
*ARS-BFGL-NGS-10830*	*rs109014211*	1.74E-09	21	14303664	*SLCO3A1*
*BovineHD2200009526*	*rs110064285*	8.84E-09	22	33753508	*FAM19A1*
*BovineHD2200009645*	*rs135018045*	3.20E-08	22	34006051	*FAM19A1*
*BovineHD2200009658*	*rs133223316*	5.38E-09	22	34051778	*FAM19A1*
*BovineHD2300014695*	*rs110724706*	4.60E-09	23	50469508	*TUBB2B*
*BovineHD2500003334*	*rs42064606*	1.77E-08	25	13011549	*SHISA9*
*BovineHD2500003336*	*rs109087355*	2.45E-09	25	13017281	*SHISA9*

Genes and SNPs location as in the Btau4.6.1 assembly; gene symbol as in GenBank.

The *BovineHD0900019961 (rs136413030)* SNP was associated to the *VNN1* (*vanin 1*) on BTA 9, the *BovineHD1500008135 (rs134980659)* SNP was associated to the *THY1* (*Thy-1 cell surface antigen*) located on BTA 15 and the *BovineHD2100001405 (rs133992914)* SNP was associated to the *IGF1R (insulin-like growth factor 1 receptor)*, located on BTA 21.

Also the *BovineHD1500008366 (rs41754552)* and the *BovineHD1500008367 (rs110269361)* SNPs were located respectively at 594,104 bp and 601,630 bp from *THY1* on BTA 15*.*

*THY1* is one of the genes differentially expressed between control quarters from cows infected with *E. coli* and *S. aureus* pathogens [11]. Also Moyes et al. 2010 [12] reported the *THY1* upregulation in *S. uberis* intramammary infections.

Sugimoto and Sugimoto [13] provided evidence that the *IGF1R* is involved in innate immunity through autophagy (general term for the degradation of cytoplasmic components within lysosomes, [14]) in bovine. In *Bos taurus*, in fact, a polymorphism in the 5′UTR region of *IGF1R* (BTA 21) was associated to mastitis incidence, determining the inhibition of autophagy in response to *S. Agalactiae* invasion.

### Nearby genes SNPs

The *BovineHD0900019716* (*rs109049649*), the *BovineHD4100007550* (*rs41662465*) and the *Hapmap49339-BTA-84110 (rs41662464)* SNPs were mapped near the *VNN1* (*vanin 1*) and the *VNN2* (*vanin 2*) located on BTA 9 respectively at 73.37 Mb and 73.39 Mb. On the same BTA 9, the *BovineHD0900019961* (*rs136413030*) SNP was close to *VNN2*. Jiang et al. [15] reported that *VNN1* and *VNN2* are related to resistance to bovine mastitis, being ranked among the 160 most mastitis relevant genes.

On BTA 19, at 55 Mb, *SOCS3* (*suppressor of cytokine signalling 3*) was found at 673,863 bp upstream the *BovineHD1900015066 (rs132720248)* SNP. This gene, important for the mammary tissue homeostasis, encodes an intracellular inhibitor of cytokine signaling, thus playing an important role in the initial steps of the recognition of pathogen-associated molecular pattern (PAMP) of the innate immune cells. This leads to the activation and initiation of the innate and the adaptive immune responses. Heeg and Dalpke [16] and Brenaut et al. [17] found the *SOCS3* gene among the 39 differentially expressed genes in milk fat globules of goats in response to an experimental intramammary infection with *S. aureus.*

The gene encoding for the *serine dehydratase* (*SDS*) on BTA 17 was located 416,619 bp upstream of the *BovineHD1700018352 (rs135157738)* SNP. This gene is included in the glycine, serine and threonine metabolism, as reported by [18]. These authors demonstrated that the serine dehydratase is one of the enzymes that changed significantly in bovine affected to mastitis.

Four SNPs on BTA 9 (*BovineHD0900019961* (*rs136413030*), *BovineHD0900019716* (*rs109049649*), *BovineHD4100007550* (*rs41662465*) and *Hapmap49339-BTA-84110* (*rs41662464*)) mapped near *CTGF (connective tissue growth factor*). *The ZNFX1 (X1-type zinc finger-containing*) on BTA 13 was close to four SNPs (*BovineHD4100010442* (*rs41634068*)*, BovineHD1300022626 (rs137320993), BovineHD1300022630 (rs109123247) and BovineHD1300022672 (rs41710487)*). The *TRIM21* (*tripartite motif containing 21*) was located 444,354 Mb upstream the strongest association chromosome region identified in BTA 15 (Table 3). The *CXCL2 (Chemokine (C-X-C motif) ligand 2)* and the *CXCL10 (Chemokine (C-X-C motif) ligand 10)* on BTA6 were significantly associated to the *BovineHD0600025253* (*rs42615160*) SNP.

**Table 3:** **List of chromosome regions strongly associated to SCS**

**BTA**	Start*	End*	**Lenght (BP)**	**N. SNPs**	**Genbank SNP code**
1	21625461	21632949	7488	3	*rs110141424; rs42365792; rs42367069*
1	27814460	28017039	202579	7	*rs135454183; rs110174548; rs134436790; rs136371716; rs111001290; rs41586446; rs110002182*
2	117668432	118739748	1071316	9	*rs134103593; rs109545959; rs133621389; rs135143470; rs136343471; rs109908642; rs133815275; rs135205101; rs43320680*
3	6388643	6396280	7637	3	*rs110787209; rs42458782; rs132773940*
4	117852857	118898784	1045927	4	*rs133335423; rs43417362; rs133867064; rs136879377*
9	72784616	72804256	19640	4	*rs41662464; rs109049649; rs41662465; rs136413030*
13	78273095	78416778	143683	4	*rs41634068; rs137320993; rs109123247; rs41710487*
15	28399876	28999494	599618	3	*rs134980659; rs41754552; rs110269361*
15	31285729	32027462	741733	5	*rs135835073; rs29018094; rs110325464; rs43299708; rs43299703*
15	50438721	51638163	1199442	14	*rs137687321; rs108941833; rs41769292; rs134338365; rs41769258; rs41769237; rs109649273; rs41768429; rs41768423; rs41768414; rs41768364; rs109966062; rs41768379; rs136525289*
17	67344705	67375670	30965	3	*rs110644998; rs134453171; rs41850009*
21^#^	60154246	60175026	20780	4	*rs29018575; rs42236250; rs42236274; rs109897238*
22	33753508	34051778	298270	3	*rs110064285; rs135018045; rs133223316*

Start. End*: candidate region start and end (bp).

^#^Start and End position referred to Btau4.6.1 assembly.

The genes above mentioned near to significant SNPs (*ZNFX1*, *CTGF*, *TRIM21*, *CXCL2* and *CXCL10)* are significantly differentially expressed by the bovine mammary epithelial cells stimulated with *E. coli* crude lipopolysaccharide [19].

Jensen et al. 2013 [11] studied and compared the transcriptional responses of uninfected mammary gland quarters adjacent to quarters infected with *E. coli* and *S. aureus* in Holstein cows*.* The *CXCL2* resulted to be one of the genes differentially expressed between control quarters infected with both the pathogens, while the *CXCL10* resulted to be one of the genes differentially expressed in control quarters from animals infected with *S. aureus* for 24 and 72 hours.

The *BovineHD2200003506* (*rs110821186*) SNP on BTA 22 mapped close to the *MYD88 (myeloid differentiation primary-response gene 88)* at 11.72 Mb, which plays a functional role in transducing pro-inflammatory molecule lipopolysaccharide (LPS) that are responsible for the majority of acute clinical cases of mastitis [20].

### Chromosome regions associated to SCS and clinical mastitis

Table 3 reported a list of the chromosome regions defined by at least three SNPs that were strongly associated to SCS. The highest number of significant SNPs (14) exceeding the significant threshold for genome-wide significance signal was found on BTA 15 (located at 50.43-51.63 Mb). On the same BTA 15, also two smaller peaks consisting of three SNPs located at 28.39-28.99 and 5 SNPs located at 31.28-32.02 Mb were identified. These regions are located in QTL that were mapped, respectively, for clinical mastitis using a linkage analysis [21] and for SCS [22]. The region located at 50.43-51.63 Mb on BTA 15 has not been reported before in cattle breeds (http://www.animalgenome.org/cgi-bin/QTLdb/index), thus identifying a supposed candidate chromosome region associated to SCS. The chromosome region on BTA 9 (72.78-72.80 Mb) mapped in a QTL region previously identified for the general disease resistance (including clinical mastitis) and for SCS [23].

Lund et al. [21] found a QTL region associated to SCS located at 32.62-43.31 Mb on BTA 22. In our study, three significant SNPs were in this region.

Sahana et al. [24] in a study on the confirmation and fine-mapping of clinical mastitis and SCS QTL in Nordic Holstein cattle using BovineSNP50 BeadChip, found the highest number of significant associations on BTA 6 identifying a QTL region for clinical mastitis at 83.37-88.89 Mb (UMD3.1 assembly). This result was also confirmed in a recent study in German Holstein cattle [25]. In our study, two significant SNPs (*BovineHD0600023179* (*rs133319155*) and *BovineHD0600023185* (*rs136907262*)) were found respectively at 84.25 and 84.26 Mb on BTA 6 (UMD3.1 assembly; Btau4.6.1 assembly position was not available), being mapped within the QTL region described by the authors previously cited (see Additional file 1).

### Annotation

Among the 36 genes listed in Table 2, the annotation data were available for 23 genes reported in the Additional file 2. This lists the biological processes (BP), the cellular components (CC), the molecular function (MF) and the metabolic pathways (KEGG) obtained with the annotation analyses performed with DAVID online Database.

The literature brings evidence that some of the genes reported in Table 2 map in QTL associated to traits of economic importance in bovine (http://www.animalgenome.org/cgi-bin/QTLdb/BT/index), as showed in Additional file 3. Those mapping in QTL already associated to clinical mastitis and SCS reported in the QTLdb were only 4: the *PLXNA4 (plexin A4)* on BTA 4, the *THY1 (Thy-1 cell surface antigen)* on BTA 15 and the *SHISA9 (known as CKAMP44, shisa homolog 9)* on BTA 25, the *FAM19A1* (*family with sequence similarity 19 (chemokine (C-C motif)-like), member A1)* on BTA 22 associated with SCS. This study thus highlighted possible QTL related to mastitis resistance in the other 19 genes annotated and considered in the GO analysis.

## Conclusions

This is the first mapping for SCS in Valdostana Red Pied population, an autochthonous alpine dual purpose cattle breed whose selection is mainly focused on milk quality, meat production and functionality.

This study brings evidence of significant associations between SCS and SNP markers on several chromosomes in known and newly disclosed QTL regions. Some genes involved in mastitis resistance or variation of SCS content were in QTL on BTAs 9, 13, 15, 17, 19, 21, 22. In particular, the strongest associations were highlighted on BTA 15 with a total of 24 significant SNPs distributed in three regions.

The detection of genomic regions will help to understand which potential candidate genes may be responsible for the genetic variation in mastitis resistance/susceptibility, a trait of primary importance in dairy cattle breeding and farming.

## Methods

### Sampling

The Valdostana Red Pied cattle is the most common autochthonous dual purpose breed in the region Val d’Aosta (13,000 animals in 2013, almost all of them registered in the Herd Book), coming from the red pied cattle and dating back to the end of the fifth century. The National Association of Valdostana Breeders (A.N.A.Bo.Ra.Va.) provided commercial semen samples for 373 bulls and 725,337 test day records from milk routine recording from 45,410 cows. No animals were involved in the work.

The daily SCC were transformed into SCS [26]. Genetic parameters and estimated breeding values (EBVs) were calculated with a test day repeatability model on first parity cows. The model of analysis considered the fixed effects of days in milk (10 classes of 30 days each), herd-test day effect (32,870 levels), month of calving and age at calving (12 classes). Additive genetic and permanent environmental effects were considered as random. Three generations of ancestors were used for each individual extracting information from the National Herd Book for a total of 35,803 animals. Variance component estimations were calculated based on 258,680 test day records with the software VCE [27] and individual EBVs were obtained with the package BLUPF90 [28]. Deregressed proofs (DP-EBV) were calculated for 2,417 bulls according to [29].

### Pool constitution

The bull families structure was verified in terms of number of sons per bull, in order to avoid overrepresentation of a single sire. Only 1 bull had 6 sons, 4 bulls had 5 sons, 3 bulls had 4 sons and the rest of bulls had 3 or less sons. The sires were ranked according to DP-EBVs for SCS: the top 20% and bottom 20% sires were identified for the constitution of independent pools within tail of the DP-EBV distribution. In order to obtain two independent groups of different animals within tail with comparable phenotypic value, the selected samples for each tail were clustered (even and odds numbers) into 2 sub-pools.

A total of 79 samples were selected for the pools constitution as follows: 2 independent pools of 20 individuals each in the high tail and 2 independent pools of 20 and 19 individuals each in the low tail. Furthermore, for each pool, 2 DNA duplicate-pools were independently constructed from identical samples. Thus, a total of 4 pools per tail were produced.

### DNA extraction and genotyping

Bulls DNA was extracted from semen samples using the ZR Genomic DNA™ Tissue MiniPrep (Zymo). The quality control was performed on each sample to verify the DNA integrity on Invitrogen E-Gel 1% Agarose Gel. The GloMax®-Multi Detection System instrument using the Quant-iT™ dsDNA Broad-Range (BR) Assay Kit (Life Technologies), determined the initial DNA concentrations. The DNA concentration for a single sample was evaluated three times and each read was verified twice (e.g. 2 instrument runs). Samples having concentration diverging ±1 SD from the mean value were not included in the pools. Samples of DNA were normalized to a concentration of 10 ng/ul, which was reconfirmed with the same methods above described. DNA pools were constructed by taking equivalent amounts of DNA from each sample.

The final pools were concentrated to 50 ng/ul, as required for the Illumina array protocol. Each sub-pool was genotyped 3 times on different chips (array replicates). In all, 24 different chip positions on 3 microarrays were used for the pooled genotyping. Genotyping was performed using the Illumina BovineHD BeadChip (777,962 SNPs) according to the Infinium protocol. SNPs positions were accordingly to the UMB 3.1 bovine assembly.

### Statistical analysis of pools

Pools were analysed according to the SDP approach. The B-allele frequencies being a good estimator of the allele frequency of the individuals in a pool for each array replicate [30], were used in the analyses after obtaining them from the self-normalization algorithm of Illumina BeadStudio software®.

### The multiple marker test

A pipeline in R software (http://www.r-project.org/) was adapted from [31] and [32] to perform a multiple marker test. The test statistic used for each SNP was:

$$ \mathrm{Ztest} = \mathrm{D}\mathrm{test}/\mathrm{S}\mathrm{D}\left(\mathrm{Dnull}\right) $$

where Dtest is the difference of the B-allele frequencies means among tails; Dnull is the difference of the B-allele frequencies means within tails. The test statistic was distributed as *χ*^2^ with one degree of freedom under the null hypothesis of equal allele frequencies.

### Quality control

We performed the analysis after excluding the 1% of SNPs that showed the highest variability as indicated by the size of the mean measures from the replicate array within tail [9]. In addition, the monomorphic SNPs were deleted from the dataset. Anderson-Darling, Shapiro-Wilk and Kolmogorov-Smirnov normality tests were performed on the Dnull distribution [33-35].

The distribution of the p-values using the quantile-quantile (Q-Q) plot was examined to estimate the number and the magnitude of the observed associations between genotyped SNPs and DP-EBVs, compared to the statistics expected under the null hypothesis of no association.

Using the -log10 of the linkage test p-values for each SNP, a Manhattan plot was created. Manhattan plot is a SNP set out across the chromosomes for left to the right, and the heights correspond to the strength of the associations of the trait.

Bonferroni correction for multiple testing was applied in the analysis. The genome-wide significance threshold was set as a corrected p-value ≤ 0.05, which equated to a nominal p-value of approximately 7.62 × 10^−8^.

### Annotation

The annotation analysis of significant SNPs was performed using UCSC, NCBI ENSEMBL and the Bovine SNP Annotation Tool (Snat), integrating the information from a variety of public bioinformatics databases (NCBI Entrez Gene, UniProt, Gene Ontology (GO), KEGG PATHWAY and AnimalQTLdb [36]). The Illumina BovineHD SNPs positions were converted from Bos_taurus_UMD_3.1 to Btau_4.6.1 assembly using the Batch Coordinate Conversion option in UCSC database as required by Snat tools. UCSC and NCBI databases were used to annotate those SNPs not included in Snat and to verify which of the significant SNPs were close (within 1 Mb [31,37]) to functional genes. GO and pathway analyses were performed using the Database for Annotation, Visualization and Integrated Discovery (DAVID) v6.7.

## Additional files

Additional file 1:
**List of the significant SNPs identified in the Valdostana Red Pied breed.**
Additional file 2:
**List of the biological processes, cellular components, molecular function and metabolic pathways obtained with the annotation analyses performed with DAVID online Database.**
Additional file 3:
**List of the genes mapping in QTL associated to traits of economic importance in bovine.**
